# Scale‐dependent intraspecific competition of Taurus cedar (*Cedrus libani* A. Rich.) saplings in the Southern Turkey

**DOI:** 10.1002/ece3.5757

**Published:** 2019-10-29

**Authors:** Osman Yalçın Yılmaz, Ali Kavgacı, Orhan Sevgi, Erdal Örtel, Hüseyin Barış Tecimen, Abdurrahman Çobanoğlu, İsmet Yeşil

**Affiliations:** ^1^ Department of Forest Engineering Faculty of Forestry Istanbul University‐Cerrahpasa Istanbul Turkey; ^2^ Southwest Anatolia Forest Research Institute Antalya Turkey; ^3^ Department of Soil Science and Ecology Faculty of Forestry Istanbul University‐Cerrahpasa Istanbul Turkey; ^4^ Aegean Forestry Research Institute Urla Turkey; ^5^ Eskişehir Regional Directorate of Forestry Eskisehir Turkey

**Keywords:** *Cedrus libani*, intraspecific competition, mark correlation function, sapling, spatial interaction

## Abstract

Better understanding of the competitive interaction at the early development stages of the stand is crucial to help schedule silvicultural treatments for young stands and for the better management of the future stands. We used scale‐dependent analysis to improve our understanding of sapling dynamics in the pure Taurus cedar (*Cedrus libani* A. Rich.) stands in Southern Turkey. Using data from nine plots established at the western Taurus Mountains, diameter, height, and crown radii of saplings were compared, and spatial point pattern analyses were performed. We found significant differences for the mean diameter and height, and crown radii of saplings among the plots. Univariate pair correlation function showed that sapling pattern was regular only at small scales (*r* < 0.4 m) but was predominantly random. Bivariate pair correlation function revealed no evidence of spatial interaction between tall saplings and short saplings. Univariate mark correlation function revealed that strong intraspecific competition was detected at small scales (up to 0.55 m). This distance is reasonable for the juvenile age tending of Taurus cedar saplings and should be under consideration during silvicultural treatments to use the site productivity more efficiently.

## Graphical abstract

To understand and measure the intraspecific competition of *Cedrus libani* saplings, we used scale‐dependent analysis to explore this interaction. This is significant because we could not find any previous work on this conifer species.

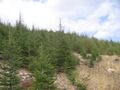

## 1 INTRODUCTION

Competition between species or individuals within a stand starts immediately following the stand initiation stage. This is promoted not only by genetic inheritance but also by environmental factors (Morgenstern, 1996). Intense competition between saplings occurs for a variety of resources including light, water, nutrients, and physical space to survive. Interactions between neighbors affect each sapling and thus the stand structure (Schneider, Law, & Illian, 2006; Vogt, Murrell, & Stoll, 2010). Understanding the outcome of competition within a stand is therefore of a critical importance for sustainable forest management, especially from a silvicultural aspect (Olivier, Robert, & Fournier, 2016). Forest managers should successfully manage the interspecific and intraspecific competition to obtain optimum wood productivity (Larocque et al., 2012) and quality (Larson, 1969). Within this context, tending operations are necessary to balance the competitive dynamics within a stand to strengthen the forest structure.

Scale‐dependent analysis is now popular in forestry studies due to its informative and directive features for forest management (Getzin, Wiegand, Schumacher, & Gougeon, 2008; Li, Ye, Hui, Hu, & Zhao, 2014; Pommerening, 2002; Stoyan & Penttinen, 2000). Spatial point pattern analysis not only shows the stand structures in detail and explains the ecological relationships, but also give information about the processes operated in the past and form a template on which processes will take place in the future (Law et al., 2009). Spatial point pattern of a stand is not only important for the latter development stages of the stand but also has crucial roles for the ecosystem dynamics (Aguirre, Hui, Gadow, & Jimenez, 2003; Goreaud, Loreau, & Millier, 2002). The current literature on spatial point pattern and competition of individuals within a stand is mainly focused on mature stands (Biber & Weyerhaeuser, 1998; Eichhorn, 2010; Fard, Feghhi, Zobeiri, & Namiranian, 2008; Getzin et al., 2006; He & Duncan, 2000; Li, Wei, Huang, Ye, & Cao, 2008; Montes, Cañellas, Río, Calama, & Montero, 2004; Pretzsch, 1997; Sekretenko & Gavrikov, 1998; Wiegand, Gunatilleke, Gunatilleke, & Okuda, 2007), but young stands are usually neglected, since tree mapping for young stands is tedious work. However, the spatial point pattern and competition at the initial stages determine the stand dynamics and structures at the later development stages. Studying competition by nonclassical methods can explain the stand spatial structure and therefore provides more information to guide tending (Hui et al., 2018; Hui, Zhang, Zhao, & Yang, 2019; Li et al., 2014).

In addition to the reduced distribution of Taurus cedar at Lebonan and Syria (Hajar et al., 2010), it is widely distributed at the Taurus Mountain line in Southern Turkey with an area of 482,000 ha (Anonim, 2015). The main distribution range of these forests is between 1,000 and 2,000 m (Kavgacı & Čarni, 2012). Due to this elevation range, these forest are not subjected to forest fires as often as Turkish red pine (*Pinus brutia* Ten.) forests, which are the characteristic fire‐prone ecosystems of thermo‐ and meso‐mediterranean belt of Eastern Mediterranean Basin (Kavgacı, Örtel, Torres, & Safford, 2016). Taurus cedar mainly appears on karstic bedrock (limestone) with superficial and deep rockiness containing cracks going down to the ground (Boydak, 2003). Taurus cedar occurs in pure stands or is mixed with *Abies cilicica*, *Pinus nigra*, *P. brutia*, *Juniperus excelsa*, *J. foetidissima*, and *Quercus* spp. (Kavgacı & Čarni, 2012).

**Figure 1:**
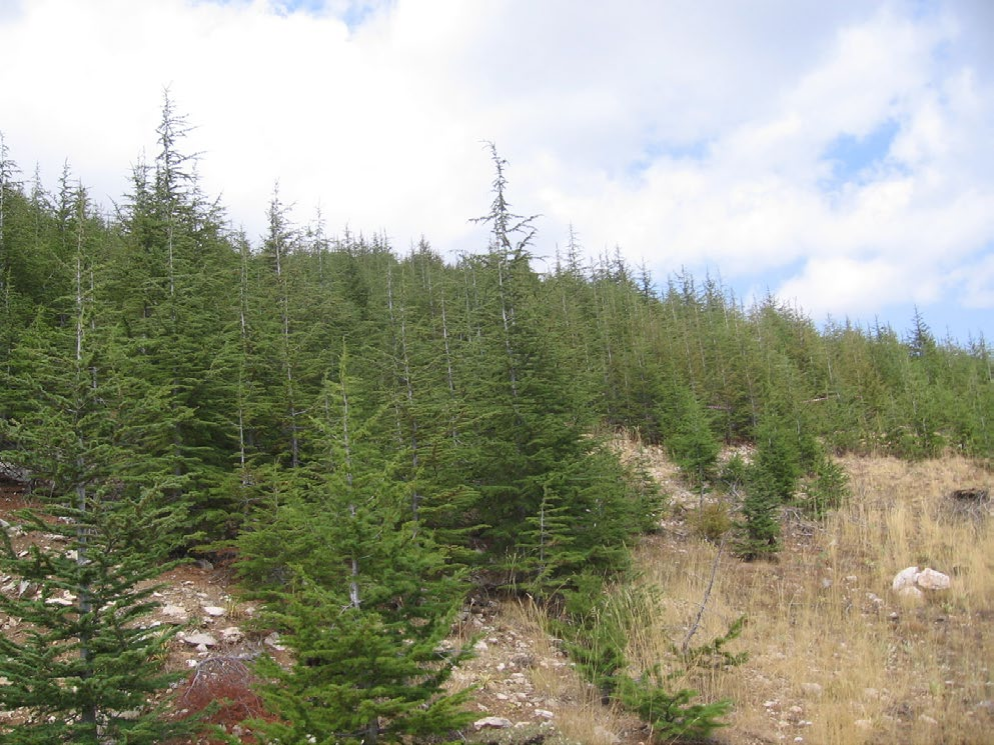
Taurus cedar saplings. Photographed in Sütleğen, Turkey, August 2015

The knowledge on the competitive relationships between Taurus cedar saplings (Figure 1) in young stands is too limited. Although some earlier studies have been conducted in young Taurus cedar stands (Eler, Özçelik, Özdemir, & Çatal, 2004; Özçelik & Eler, 2009; Yılmaz et al., 2010), to understand the effects of cuttings with different intensities on tree growth, these forest growth and yield experiments were not scale‐dependent and missing precise descriptions on the relations based on tree spatial position and tree size properties (e.g., diameter breast height, height, crown width) (Pretzsch, 2009). There is generally a lack of information on the spatial structure of Taurus cedar stands despite the ecological and economic importance of this species.

This study, therefore, attempted to address this gap and aimed to understand the spatial point pattern of pure young Taurus cedar stands and scale‐dependent competitive interactions within the stands, which would be useful for early‐stage silvicultural treatments. Accordingly, the objective of this study was to answer the following specific questions: (a) How do size attributes change between cedar saplings? (b) Does local crowding reduce plant growth? (c) How do these interactions differ by site class? (d) Is there an intraspecific competition between cedar individuals as expected? Therefore, are there any cleaning operations required for these cohorts? (e) To what scale are these interactions lasting?

## 2 MATERIALS AND METHODS

### 2.1 Study area

**Figure 2:**
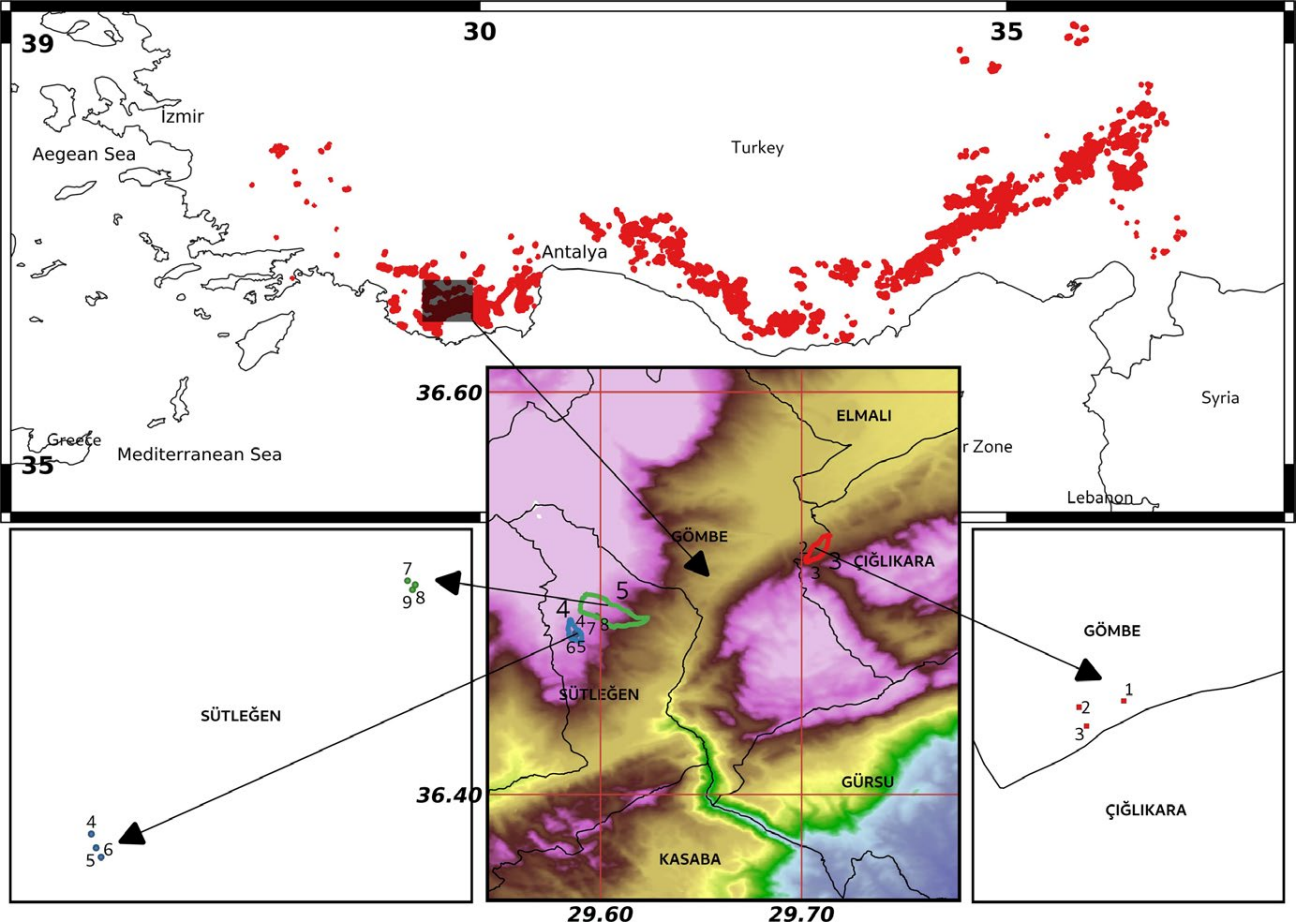
Maps showing the distribution of Taurus cedar stands in Southern Turkey (red points on the main map) and the location of the nine sample plots in the three site classes are shown on the color‐sliced elevation map (red: III site class, blue: IV site class, green: V site class)

The study was conducted at three pure Taurus cedar stands at the sapling stage in the western Taurus Mountains in Southern Turkey, which represents one of the densest distributions of Taurus cedar (Kavgacı, Başaran, & Başaran, 2010). The study sites are located between 36.40 and 36.60 northern longitudes and 29.50 and 29.80 western latitudes (Figure 2) and are located mainly on south‐facing slopes (Table 1) with a dry habitat. The altitude of the research area is 1,550–1,880 m above sea level. According to the Thornthwaite classification system, this region has a humid microthermal continental climate, which is close to oceanic climate (Başaran et al., 2008). Water deficiency is present during the summer from the middle of July to the middle of September. The annual total precipitation is 725.4 mm. The highest amount of precipitation occurs in December with 134.3 mm while it is only 9.1 mm in August. The annual mean temperature is 7.1°C. The hottest month in the year is July with 18.0°C while the coldest one is January with −2.3°C. The bedrock is mainly limestone, and the soil texture is loamy clay or clay (Kavgacı et al., 2010). The study area is formed by karstic land with shallow or medium soil depth and cracked bedrock (Boydak & Çalıkoğlu, 2008). This site conditions let the roots easily develop and penetrate along the cracks in the porous, well‐drained limestone rocks, which results in a patchy natural regeneration pattern instead of an uninterrupted regeneration mass (Boydak, 2003; Kavgacı et al., 2010). Seeding is therefore a more successful reforestation technique for these bare karstic lands. Planting following traditional mechanical site preparation is not suitable on these sites with hard soil conditions (Boydak & Çalıkoğlu, 2008).

**Table 1:** Stand characteristics of the nine Taurus cedar plots in the three site class

Plot	P1	P2	P3	P4	P5	P6	P7	P8	P9
Site class	III			IV			V		
Stand age	28			27			29		
Elevation (m.a.s.l.)	1,578	1,565	1,560	1,867	1,859	1,879	1,824	1,823	1,834
Aspect	SE	ES	ES	SW	SW	SW	E	E	E
Slope (degree)	10.10	10.74	10.17	13.39	15.03	17.08	12.47	12.96	12.91
Latitude	29.70479	29.70397	29.70411	29.58752	29.58768	29.58785	29.59813	29.59839	29.59830
Longitude	36.51678	36.51667	36.51632	36.48011	36.47965	36.47934	36.48861	36.48848	36.48831
Stocking (saplings per ha)									
TS	14,800	10,600	19,900	8,100	13,500	10,300	10,300	11,800	12,400
SS	2,800	1,700	5,100	1,700	2,900	1,900	1,600	2,700	1,800
AS (total)	17,600	12,300	25,000	9,800	16,400	12,200	11,900	14,500	14,200
Cross‐sectional area of mid‐diameter (m^2^ per ha)									
TS	16.41	14.33	12.20	23.67	16.09	22.33	11.91	13.16	9.98
SS	0.13	0.10	0.07	0.30	0.04	0.07	0.07	0.11	0.05
Height (m)									
Mean stand height	1.94	2.15	1.32	3.01	1.77	2.53	1.62	1.54	1.29
TS (mean)	2.19	2.39	1.56	3.52	2.08	2.92	1.80	1.80	1.42
TS (*SD*)	0.90	1.02	0.70	1.12	0.84	1.04	0.62	0.63	0.50
SS (mean)	0.63	0.68	0.41	0.62	0.32	0.42	0.44	0.40	0.40
SS (*SD*)	0.21	0.25	0.15	0.49	0.19	0.26	0.15	0.17	0.16
H100	4.52	4.62	3.51	5.29	3.75	4.95	2.99	3.12	2.56
Diameter at 0.30 (cm)									
TS (mean)	3.40	3.67	2.40	5.77	3.60	4.96	3.59	3.50	2.99
TS (*SD*)	1.59	1.93	1.42	1.97	1.50	1.73	1.37	1.39	1.14
SS (mean)	0.66	0.75	0.29	0.86	0.20	0.40	0.56	0.52	0.49
SS (*SD*)	0.38	0.39	0.30	1.24	0.37	0.58	0.45	0.50	0.36
Crown radius (m)									
AS (mean)	0.43	0.49	0.33	0.62	0.42	0.51	0.47	0.43	0.43
AS (*SD*)	0.20	0.23	0.19	0.28	0.21	0.23	0.18	0.20	0.14

Abbreviations: AS, all saplings; SS, short saplings; TS, tall saplings.

Taurus cedar seedlings on karstic sites grow slowly for the first 4–6 years (Kantarcı & Odabaşı, 1990), since below‐ground growth is prioritized rather than aboveground as a typical adaptive resource allocation strategy to avoid drought (Boydak, 1988; Dirik, 1998). During this period, the number of seedlings also decrease, and after that, the mortality is stabilized, and height differentiation starts (Kantarcı, 1987c). This differentiation may appear in small patches of individuals in accordance with site characteristics (Boydak & Çalıkoğlu, 2008). The multilayered stand structure is a characteristic for the release cutting stage at Taurus cedar forests at karstic lands, as in our case. The release cutting stage ends with the appearance of stem exclusion and self‐pruning. Taurus cedar is a light‐demanding tree species, but it can survive in partial shade conditions at a young age, and its main natural regeneration technique is shelterwood system that builds up an even‐aged stand (Boydak, 2003). Taurus cedar forests in Turkey had been managed with the uneven‐aged system until 1965, and later, the even‐aged system generated by shelterwood cutting has been preferred (Köse & Yavuz, 1990). The seedlings reach to the sapling stage at ages of 10–15 years. Without any silvicultural interventions, groups with height differences appear in the stand. Boydak (2003) stated that the growth performances of Taurus cedar individuals are largely determined by root competition and likelihood of an individual finding a microsite with a crack in the bedrock for root growth. At stands with no silvicultural intervention, stand density may reach up to 20,000 seedlings/ha (Kantarcı & Odabaşı, 1990).

### 2.2 Data

**Figure 3:**
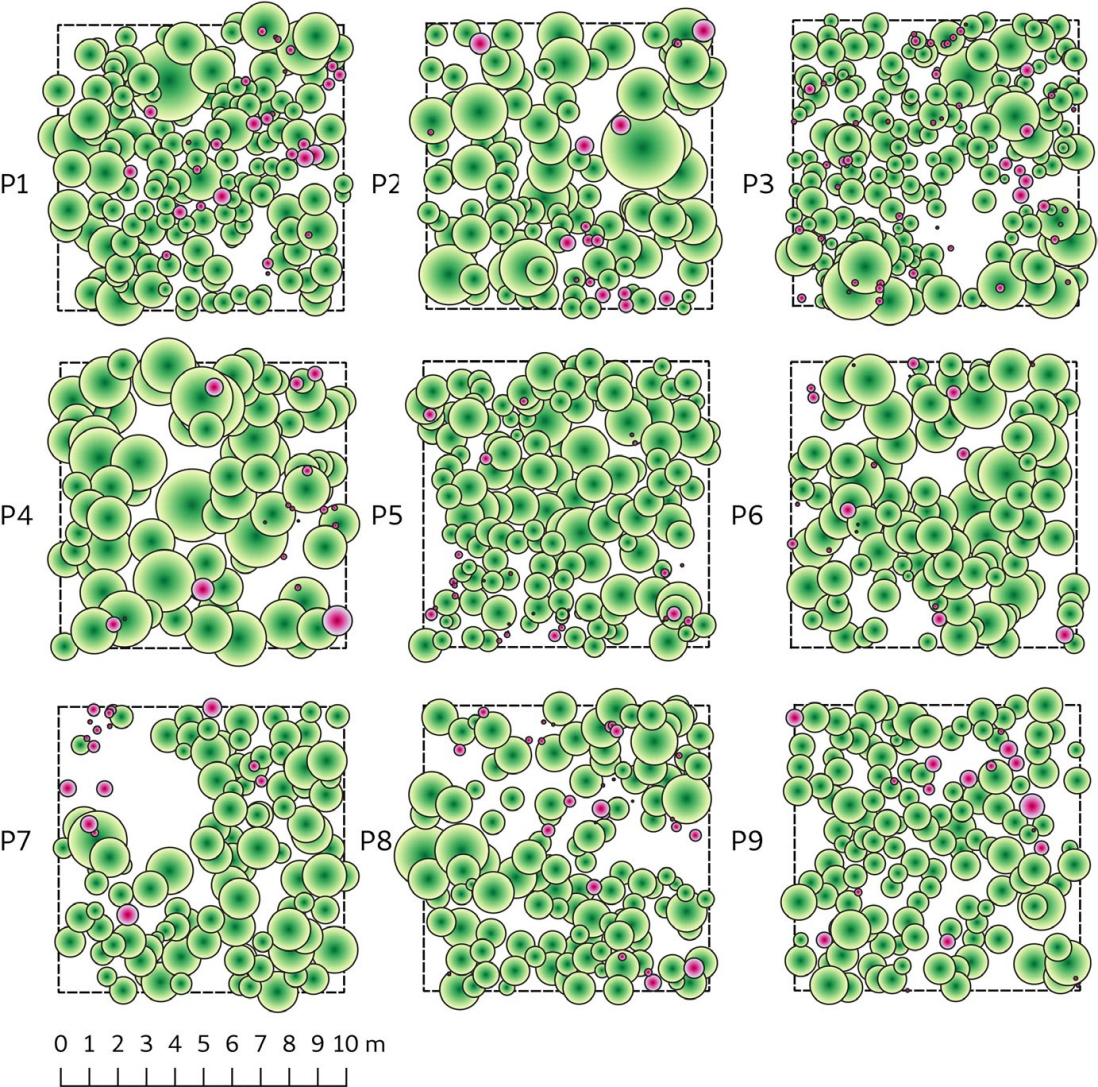
Spatial distribution of the Taurus cedar tall saplings, that is, saplings with height exceeding the stand mean height (green circles) and short saplings, that is, saplings with height not exceeding the stand mean height (purple circles) individuals in the sampling plots. Crown coverages were used for projection. P1, P2, P3, P4, P5, P6, P7, P8, and P9 represent the plot numbers

Three different sites which are at the III, IV, and V site class were chosen to test the site quality effects on spatial distribution and competitive interactions of Taurus cedar saplings. These site quality values, average height of the dominant trees at one hundred years of age, were taken from the historical forest management plans of the relevant forest districts. All stands, which were naturally regenerated by shelterwood system, were at sapling stage and pure. In August and September 2014, we established three 13 m × 13 m rectangular plots for each site class. During the plot establishment, we excluded the areas without saplings due to patchy regeneration pattern (Boydak, 2003) and paid a special attention to minimize topographic variation within the plots. All living saplings were surveyed using a total station (Figure 3). In the plots, there were not any mature trees larger than the saplings present. Stem diameter at 30 cm (hereafter mid‐diameter: MD) and 130 cm (diameter at breast height: dbh) aboveground (where present) was measured with a caliper with a precision of 1 mm. All individuals' total heights were measured with a measuring pole at 1 cm accuracy. Actual crown diameter was measured with a meter tape in two directions (parallel and perpendicular to the slope) for estimating crown radii.

### 2.3 Data analysis

The data for all saplings were measured and interpreted due to the importance of small saplings in the intraspecific competition (Shackleton, 2002). Taurus cedar all saplings (hereafter: AS) of each plot were divided into two size classes based on mean stand height of that plot (Table 1), by classifying saplings with heights above mean stand height as tall saplings (hereafter: TS) and those with heights below the mean stand height as short saplings (hereafter: SS), to better describe the stands' structure and to explain interactions. Because some of the saplings were shorter than 1.30 m, we did not use dbh for further analysis. To embrace the intrinsic competition relations and to eliminate the site quality variances, saplings were evaluated by these size classes in each plot (Petritan, Marzano, Petritan, & Lingua, 2014). Analyses were performed using R‐software (R Core Team, 2018) and QGIS (Quantum GIS Development Team, 2019).

### 2.4 Stand structure

**Figure 4:**
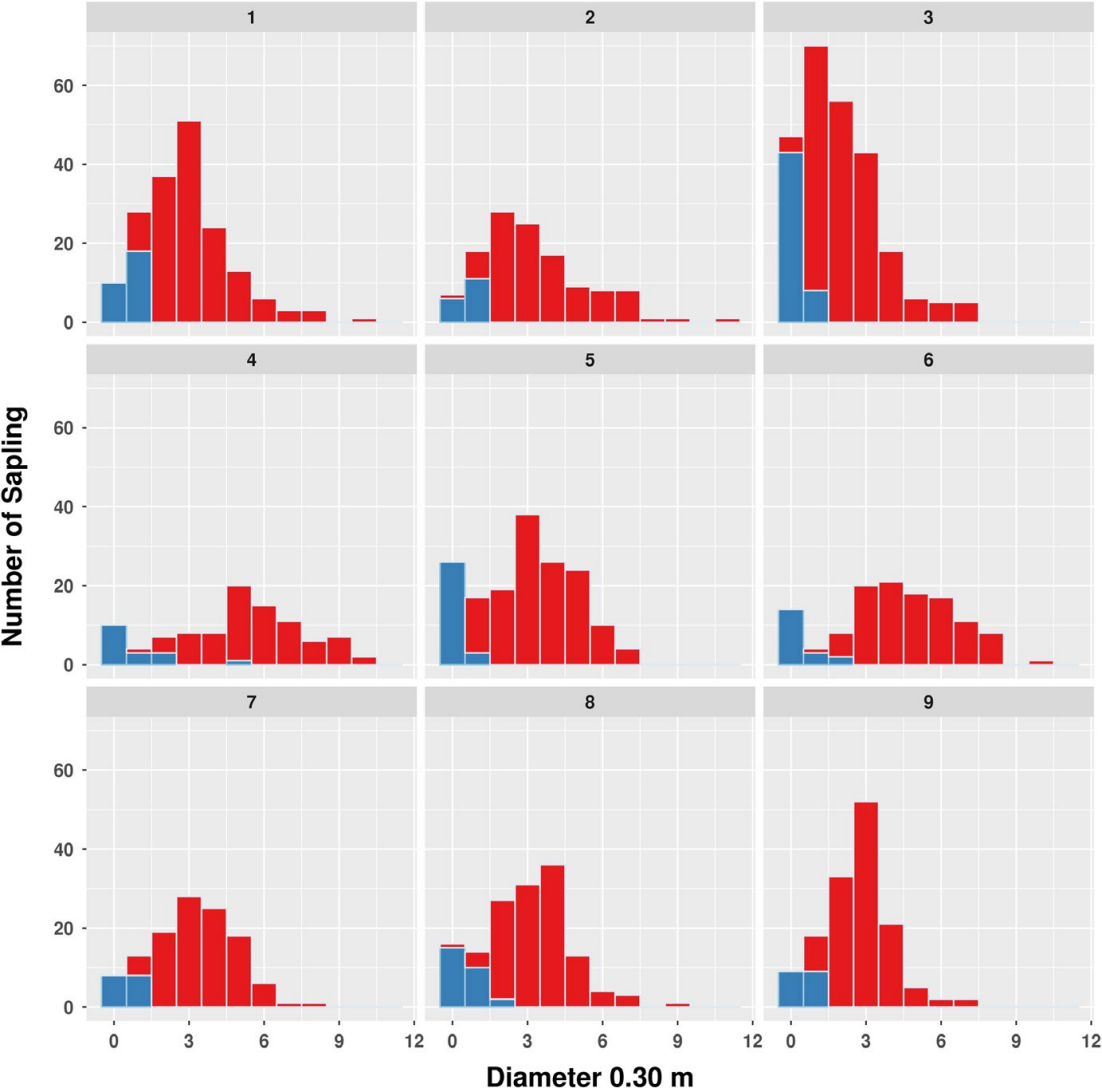
Stacked histogram graph showing the diameter distribution of tall saplings (red) and short saplings (blue) at each study plot (class bin 1 cm). 1, 2, 3, 4, 5, 6, 7, 8, and 9 represent the plot numbers

The overall frequency distributions of the TS and SS of the Taurus cedar for each diameter class were compared (Figure 4). Number of trees per hectare, total cross‐sectional area by MD, mean and standard deviation of MD, mean and standard deviation of dbh, mean height, maximum height, and dominant height (H100) were calculated (Table 1). Height, crown radius, and diameter of saplings were compared between and within three site classes. The assumptions of normality and homogeneity of variance of the residuals were examined using the one‐sample Kolmogorov–Smirnov test. Because the data distribution did not fit a normal curve, the data were subjected to several transformation methods but none of the transformations resulted in normal distribution. Therefore, using nonparametric Kruskal–Wallis and Bonferroni‐corrected Mann–Whitney *U* tests was necessary for post hoc pairwise comparisons.

### 2.5 Scale‐dependent competition

Spatial point pattern analysis was used to investigate the spatial pattern and intraspecific competition of Taurus cedar saplings. To prevent misinterpretation of the results, different hypotheses were tested using different null models (Goreaud & Pélissier, 2003; Petritan et al., 2014). First, we checked our data homogeneity with the null model of complete spatial randomness (CSR) (Wiegand & Moloney, 2004). To measure CSR, “quadrat.test” based on quadrat counts was used. The “quadrat.test” performs chi‐squared tests or Monte Carlo tests of goodness‐of‐fit for a point process model. We shifted the 10 m × 10 m rectangular sampling boundary (window of observation) in the stem mapped area (13 m × 13 m). The pair correlation function, which was used for the null model, is also sensitive to effects from heterogeneous spatial pattern (Wang, Wiegand, Hao, Li, & Lin, 2010). Second, to assess the spatial pattern of the AS and TS, we utilized the univariate pair correlation function g(r) (Stoyan & Stoyan, 1994; Wiegand & Moloney, 2004) under a homogeneous Poisson null model. To detect spatial relationships between saplings in different size classes (i.e., TS‐SS), the bivariate pair correlation functioning g_12_(r) was used. Third, the spatial correlations of the mark “crown radius” based on sapling locations in the nine plots were determined using the mark correlation function (MCF). Although other growth characteristics were measured in the study, crown radius was selected for MCF, because it better represents competition (Sharma, Vacek, & Vacek, 2016). The mark correlation function considers the quantitative characteristics (such as crown radii which is associated with sapling positions) and then calculates the spatial correlation of these marks in the observed point pattern (Wiegand & Moloney, 2014). Mark correlation function is a widely used scale‐dependent analysis technique to clarify the competitional relationships between trees (Getzin et al., 2008; Pommerening, 2002) and understanding the probable changes in the stands. Mark connection functions of the saplings in nine plots were examined to explore the bivariate pattern of tall saplings to short saplings. All univariate and bivariate point pattern analyses were performed using “spatstat” package of R‐software (Baddeley, Rubak, & Turner, 2015). For all scale‐dependent analyses, significant departure from the null models was evaluated based on 95% simulation envelopes, which were calculated from the 5th‐lowest and 5th‐highest values of 1,000 Monte Carlo simulations.

## 3 RESULTS

### 3.1 Stand structure

According to the nonparametric Kruskal–Wallis test and post hoc comparisons, values of mid‐diameter, height, and radii of saplings not only the three site classes but also nine plots differed significantly (*p* < .05). In the nine plots, there were 1,339 individuals in total and 222 of them were not taller than the mean height of all saplings (Table 1). Density of TS ranged from 8,100 to 19,900 individuals/ha while SS varied between 1,600 and 5,100 in the study sites (Table 1). We detected that the number of individuals in the plots possessing the highest density (P3) is 2.5 times higher than the plot with lowest stand density (P4). The TS cross‐sectional area of MD is the highest in P4 and lowest in P9. The diameter distribution exceeds 8 cm significantly only in P4 and P6, while the others have only a few trees with diameters above 8 cm (Figure 4). The peak of diameter distribution curve concentrates typically at 4 cm. Compared to other plots, P4 and P6 had the highest cross‐sectional area, mean height, dominant height, mean diameter which can be attributed to higher site quality and/or low density. On the other hand, P9 had the lowest cross‐sectional area, dominant height, and mean height except mean diameter because of poor site quality and/or high number of saplings. Average height of TS individuals ranged from 1.42 to 3.52 m and H100 ranged from 2.56 to 5.29 m in all plots (Table 1).

### 3.2 Scale‐dependent competition

**Figure 5:**
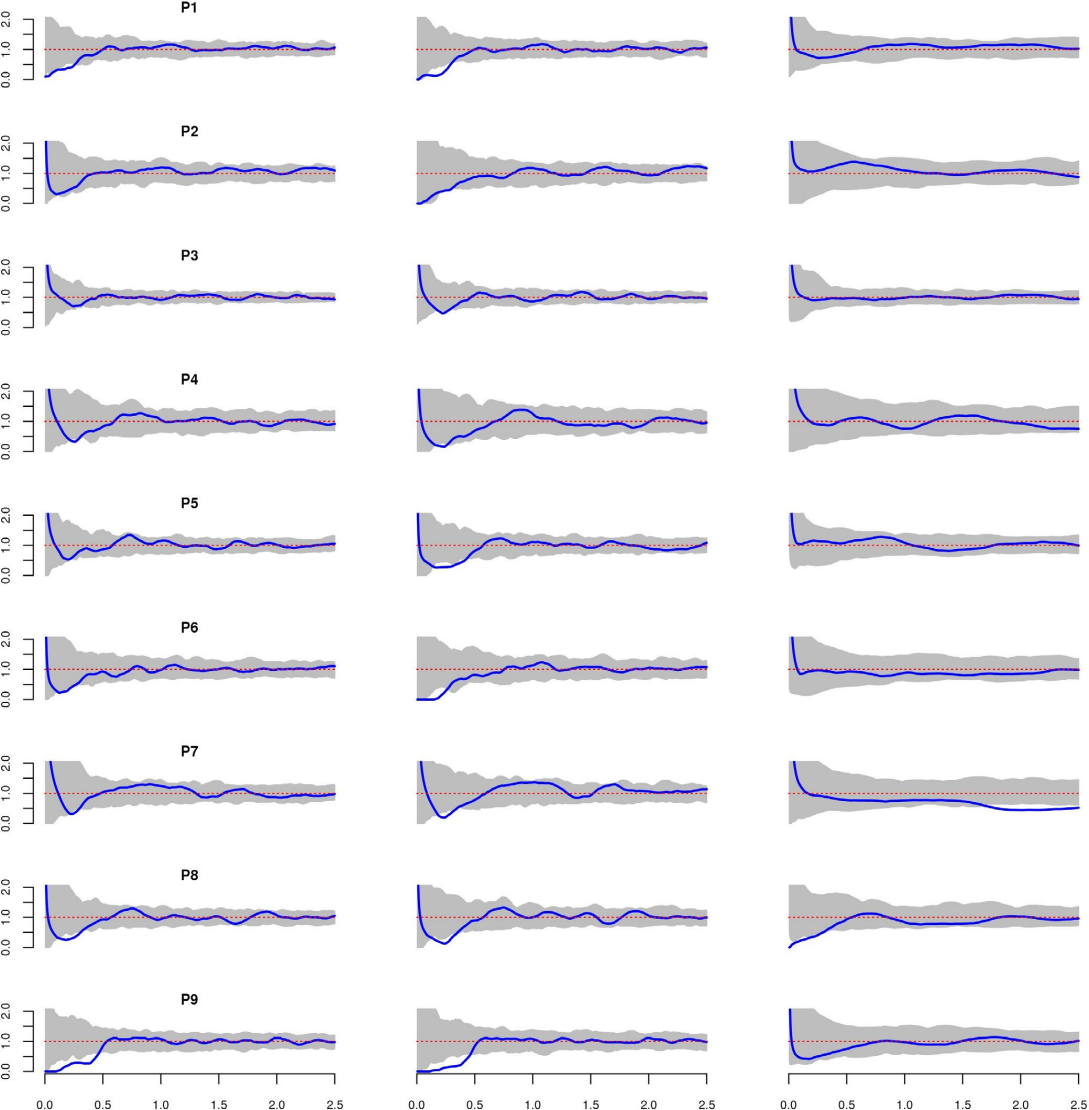
Sapling spatial patterns quantified with the pair correlation function g(r) for the all saplings (left) and for the tall saplings (middle). In the right graphs, bivariate pair correlation function g12(r) between TS and SS. Envelopes of the acceptance regions of the CSR hypothesis are shaded. *X*‐axis shows the distance in meters between pairs of saplings

The pair correlation function of all univariate and bivariate patterns approached to their asymptotic value of approximately one within 2.5 m (Figure 5) which supports the quadrat test results of the sampling plots pattern (Figure 3) by being homogeneous. But in P7 bivariate pair correlation function of TS versus SS shows significant repulsion at distance >1.5 m. The univariate pattern was mainly random for AS in P2 and P3. In other plots, the univariate AS pattern was regular only at small scale (*r* < 0.4m) but it was predominantly random. In P5, AS were randomly distributed except for the distance interval 0.6–0.7 m where saplings were clumped. The bivariate pair correlation function for TS versus SS indicated no evidence of a significant spatial interaction between TS and SS at all plots except P7.

**Figure 6:**
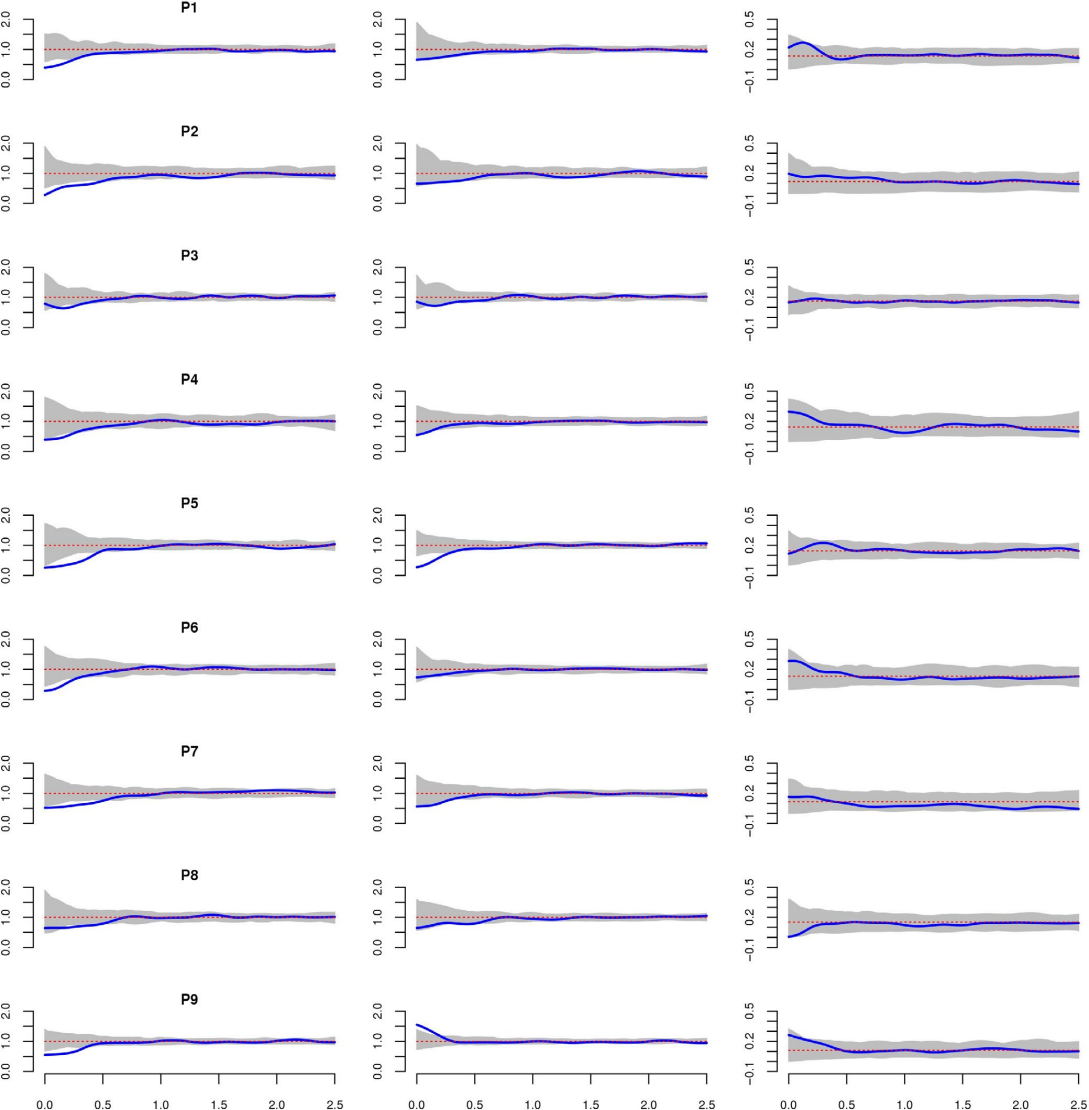
Mark correlation function, kmm(r), using crown radii as marks in all saplings (left) and tall saplings (middle). Mark connection function of TS and SS (right). *X*‐axis shows the distance in meters between pairs of saplings. Envelopes of the acceptance regions of the mark independence hypothesis are shaded

We compared scale‐dependent competition of saplings with mark correlation function applied to the mark “crown radius” for Taurus cedar saplings. For the AS, strong intraspecific competition was detected for small scales (up to *r* = 0.55 m) in all plots (Figure 6). These negatively correlated marks show stronger mutual growth reduction. Overall, mark correlation function for the AS and TS did not show a high variance. The bivariate mark connection function for TS versus SS indicated no evidence of a significant competition between TS and SS at all plots. Mark correlation function not only showed competition distance but also gave the correlation strength (i.e., competition intensity). In P7, P8, P9, and P3 negative correlation is weaker (since the values of *k* mm (*r*) are smaller) while in P1, P2, P4, P5, and P6 stronger (since the values of *k* mm (*r*) are larger).

## 4 DISCUSSION

We found that size attributes of the pure young Taurus cedar stands show large variations. These variations can be the result of two main effects: microsite differences at short distances (Boydak & Çalıkoğlu, 2008; Kantarcı, 1985) and light tolerance character of Taurus cedar (Eler, 1990; Kantarcı, 1987a). Unlike common even‐aged stand structure (i.e., single stories), Taurus cedar saplings show an irregular stand structure, and the saplings canopy does not appear to be a uniform height resulted by the microsite differences (Chen et al., 2010; Kantarcı & Odabaşı, 1990). Taurus cedar forests generally grow on karstic lands with deep cracks in the bedrock (Boydak, 2003), which causes an asymmetric competition among individuals, providing an advantage to those with easier access to the soil nutrients (Schwinning & Weiner, 1998) than the rest of competitors. Soil water content is scarce due to insufficient precipitation, high evapotranspiration, interception, and karstic structure of the bedrock with deep cracks in the Mediterranean climate zone. This water deficiency also affects the competition (Vilà & Sardans, 1999). All of these factors result in the size attribute to differ spatially. In addition to that, although Taurus cedar is a light‐demanding species (Alptekin & Çalışkan, 1996; Boydak & Çalıkoğlu, 2008), it is also defined as partly shade tolerant during the early growth stages (Odabaşı, Çalışkan, & Bozkuş, 2004). This character of the species allowed for the growth of additional generations of seedlings originating from the standing trees from previous stand, resulting in a relatively layered structure. Namely, regeneration system of mature Taurus cedar forests is based on shelterwood system. In this system, the seeding trees stand up in the stand for a while till the final clearance and continue sowing the stand and supply the additional seedling establishments (Smith, Larson, Kelty, & Ashton, 1997). This time lag between the emergence of individuals within a stand, in addition to the probable effects of microsite differences caused by cracks in the bedrock (Boydak, 2003), may result in reduced growth of individuals that establish later and give these individuals a competitive disadvantage (Kantarcı, 1987b).

We did not detect any density‐dependent mortality (i.e., self‐thinning) in any of the studied nine plots. The number of individuals per hectare at the study sites changes between 9,600 and 25,000, which conforms to the findings of Yılmaz et al. (2010) and Kantarcı and Odabaşı (1990). According to Zhang, Wei, Zhao, and Gadow (2013) when the distance between individuals decreases at stands, negative correlation appears. In our study, at the V site class (P7, P8, P9), moderately intensive competition appears at long distances, while at IV site class (P4, P6), it appears at shorter distances and with higher intensity. Therefore, this shows that site quality should be evaluated in studies on competition as a variable.

As is known, site index of a stand is determined according to the height of the tallest trees (H100) in a mature stand at the thinning development stage and the number of trees is higher at stands with better sites then poor ones. Despite all site classes having saplings of the same age, the H100 values of P4 and P6, which are in site class IV, were much higher than those of all our plots in site class III. Although site index determination is carried out for mature stands, growth characteristics of young stands can be informative for the site conditions. Site index of the study sites was obtained from the management plan of the related forest districts. But the results revealed that young stands represent different site class value than the management plan. This could be explained by the potential and realized measures of forest site quality (Pretzsch, 2009). These results point out that using the site quality values of previous plans for the newly regenerated stands may misguide the forest managers, and site index of the stands should be checked during the latter management plan revisions.

Spatial distribution patterns and size of trees are not independent from each other, and are commonly affected by competition (Getzin et al., 2006), which occurs in two ways: above‐growth for light, and below‐growth for water and nutrient. During the regeneration stage, although the light conditions are equal for all juveniles, below‐ground competition is mostly determined by microsite differences caused by bedrock conditions at Taurus cedar distribution area (Kantarcı, 1987b). As is indicated by Boydak (2003), root competition is the main driver on the growth performances of Taurus cedar individuals. The juveniles finding a crack with deep soil in the bedrock perform better growth than the others, which causes different spatial patterns from site to site which were also approved by this study. Namely, while the competition ceases at 30 cm distance in P4 and P6 having the lowest number of individuals, the negative effects end almost at 60 cm in P2, which may be the result of the high amount of individuals in high diameter classes at this site. We could not find significant interaction between TS and SS except at P7. In TS of P7, repulsion was found after 1.5 m. This could be explained with the distribution of the tall saplings. We could not find positive interaction between Taurus cedar all saplings. This could be explained by the niche similarity and the spatial distribution of saplings. Our overall univariate scale‐dependent analysis showed significant negative interactions up to 0.55 m according to mark correlation analysis of sapling radii. Beyond the mark correlation analysis, the results suggest that intraspecific competition ends at 0.55 m, and similarly, pair correlation analysis results supported regularity at small scale (*r* < 0.4 m).

## 5 CONCLUSION

In the study, Young Taurus cedar stands showed different spatial point patterns and competitive relationships due to local changes. Competition between individuals ends in distances from 25 cm to not more than 1 m. This knowledge makes valuable contribution to silvicultural treatment at young Taurus cedar stands. Therefore, it is reasonable to offer that the distance between individuals can be arranged as 50 cm for the juvenile age tending and 1 m after first precommercial thinning as an easy application while the classical principles of young stand tending for Taurus cedar should also be taken into consideration. This tending should also focus on short saplings since the Taurus cedar favors establishing single layer stands during the later stages due to its light‐demanding character. In order for this knowledge to be applied to all pure Taurus cedar stands, similar studies should be conducted in different site conditions.

## CONFLICT OF INTEREST

None declared.

## AUTHORS CONTRIBUTION

All authors contributed to study design and data collection. O.Y.Y. and O.S. conducted analysis and wrote manuscript in collaboration with A.K. and H.B.T.

## Data Availability

3D stem maps of nine plots and R scripts generated for this study will be available through the Dryad Digital Repository. https://doi.org/10.5061/dryad.fbg79cnqw.
